# Office‐based sedation‐free transnasal esophagogastroduodenoscopy with biopsies using single‐use gastroscopes: A pediatric single‐center experience

**DOI:** 10.1002/jpr3.12025

**Published:** 2023-12-20

**Authors:** Yamen Smadi, Jessina Thomas, Khaled Bittar, Hannah Norton, Joel A. Friedlander, Jeffrey Bornstein

**Affiliations:** ^1^ Eosinophilic Esophagitis Center Arnold Palmer Hospital for Children, Orlando Health Orlando Florida USA; ^2^ Center for Digestive Health and Nutrition Arnold Palmer Hospital for Children, Orlando Health Orlando Florida USA; ^3^ Nurse Care Coordinator, Eosinophilic Esophagitis Center Arnold Palmer Hospital for Children, Orlando Health Orlando Florida USA; ^4^ EvoEndo, Inc. Centennial Colorado USA

**Keywords:** eosinophilic esophagitis, EvoEndo, transnasal endoscopy

## Abstract

**Introduction:**

Unsedated transnasal endoscopy (TNE) as transnasal esophagoscopy (TN‐Eso) has emerged as a promising alternative to esophagogastroduodenoscopy (EGD) under sedation to assess eosinophilic esophagitis (EoE). We report our center's experience using single‐use gastroscopes to perform sedation‐free transnasal EGD (TN‐EGD) with biopsies in an office‐based setting.

**Methods:**

A retrospective review was performed on patients with eosinophilic esophagitis who underwent office‐based sedation‐free TNE with topical analgesia and virtual reality (VR) procedural dissociation and distraction. A sterile, single‐use, ultra‐slim 3.5 mm outer diameter, 110 cm long gastroscope with 2 mm working channel (EvoEndo) was used to perform TNE with biopsies/brushings. Data including demographics, procedure success rate, total visit time, biopsy adequacy, procedure time, procedural preference, and complications were collected.

**Results:**

Office‐based TNE was completed in 8 patients (six males, age range 11–20 years). The endoscope was advanced by an experienced transoral endoscopist successfully through the nares into stomach (transnasal esophagogastroscopy [TN‐EG]) in all subjects (100%) and into the duodenum (TN‐EGD) in seven subjects (87.5%). Biopsies were obtained from esophagus in all cases and from the stomach/duodenum in five cases. Histological assessment, esophageal brushing, disaccharidase enzyme analysis, or duodenal aspirate analysis were performed as indicated. EoE reevaluation was the primary indication to perform endoscopy in all patients. Visual and histologic findings were all adequate for assessment. There were no significant adverse events.

**Conclusion:**

Office‐based TN‐EGD with VR procedural distraction and dissociation using single‐use gastroscopes was effective to monitor EoE, gastritis, and duodenitis in a pediatric practice.

## Abstract (highlights)

**What is Known:**

- Sedation‐free esophagoscopy with virtual reality (VR) distraction and dissociation allows for endoscopic evaluation of the esophagus and enables visualization and acquisition of biopsy samples while eliminating the risks associated with sedation and anesthesia.
- Sedation‐free esophagoscopy using reusable scopes is feasible, safe, successful, and cost‐effective in pediatric populations to monitor disorders of the upper gastrointestinal tract, particularly in diseases such as eosinophilic esophagitis that often require serial endoscopies.

**What is New:**

- Single‐use, ultra‐slim gastroscopes when used in an office‐based, pediatric setting, enable sedation‐free transnasal esophagogastroduodenoscopy (TN‐EGD) with biopsies, brushings, or aspirate and is shown to be feasible, and effective.
- The success rate of TN‐EGD when performed by an experienced endoscopist newly trained in sedation‐free endoscopy is 87.5%.

## 1 INTRODUCTION

Pediatric unsedated transnasal endoscopy (TNE) has emerged as a promising alternative to sedated esophagogastroduodenoscopy (EGD) which generally requires anesthesia in children and adolescents. Sedation and general anesthesia are associated with increased adverse events, monitoring, staffing requirements, higher costs, and a longer overall procedure and recovery time.^1^, ^2^ Sedation‐free TNE is particularly useful in diseases that require repeated upper gastrointestinal tract assessments. Indications for TNE described in pediatrics include the evaluation of dysphagia, Barrett's esophagus, esophageal candidiasis, esophagitis in patients with tracheoesophageal fistula/esophageal atresia, abdominal pain, gastroesophageal reflux disease (GERD), and celiac disease.^3^, ^4^ Distraction techniques are helpful both in preparation for and during unsedated TNE. One widely used distraction technique for pediatric patients is the use of virtual reality (VR) headsets.^3^, ^5^, ^6^

Performing TNE in an office‐based setting provides benefits to the physician and the families including efficient physician workflow, adequate staffing, and avoidance of hospital or surgical center fees. More importantly, it may also provide a less threatening and more time efficient experience for patients and families. A single‐use endoscopy system helps overcome a lack of onsite facilities for sterile instrument processing.

Despite the recent increased reported use of TNE in pediatrics,^7^, ^8^ performing full sedation‐free transnasal EGD in the United States is limited.^2^ Previously, the only available ultra‐slim endoscopes built for transnasal esophagogastroduodenoscopy (TN‐EGD) of the gastrointestinal tract had a shaft diameter greater than >5.5 mm. Recently, a sterile, single‐use, ultra‐slim 3.5 mm outer diameter, 110 cm long gastroscope with a 2 mm working channel (EvoEndo) which allows the completion of a full EGD with obtaining biopsies and other necessary samples when indicated became available. It is approved for use in patients ages 5 years and older. The system includes a portable video controller, and a “patient experience kit” containing VR goggles and a stress ball for patient distraction during the procedure, allowing for an optimized experience during a sedation‐free procedure.

We report our center's initial experience with the EvoEndo system, in which eight patients underwent TNE as transnasal esophagogastroscopy (TN‐EG) and TN‐EGD with (VR) procedural dissociation and distraction in an office‐based setting instead of sedated EGD. The aim of this study was to evaluate the clinical outpatient use of sedation‐free pediatric TNE. We report the completion rate of esophageal, gastric, and duodenal intubation, total patient time in the office, adverse events (AEs), procedural preference, and adequacy of visual and histologic findings.

## 2 METHODS

### 2.1 Study design

A retrospective study of children who underwent sedation‐free TNE in an office‐based setting at a single academic pediatric medical center at Arnold Palmer Hospital for Children, Orlando, FL, USA between April and June 2023 was performed using data from the program's clinical database. EoE reevaluation was the indication for endoscopy in all patients. Local IRB reviewed the study and designated it as exempt. Data are expressed as medians and interquartile range.

### 2.2 Procedure description

Subjects were asked not to eat for 6 h and not to drink for 2 h before the TNE. In an outpatient clinic room, subjects sat in a chair designed for outpatient laryngoscopy procedures. Single‐use video goggles with YouTube‐based VR programming (EvoEndo Channel) for dissociation and distraction were applied. Topical lidocaine 2% gel was applied to the nasal passages and throat. Topical oxymetazoline was used as needed when nasal congestion was appreciated by the endoscopist. After performing the initial six cases, we thought Simethicone might decrease gastric bubbles and therefore, Simethicone 125 mg chewable tablet was given orally to decrease gastric bubbles in the last two cases. The endoscopist engaged the patient throughout the whole procedure and explained each step.

All TNEs were performed by one gastroenterologist (Yamen Smadi), and one fellow assisted for their training (Jessina Thomas). TNE was performed using a sterile, single‐use, ultra‐slim 3.5 mm outer diameter, 110 cm long gastroscope with four‐way deflection and a 2 mm working channel (EvoEndo). The patient was positioned so their face and nose were visualized approximately upright on the screen and the endoscopist adjusted the tip of the scope into a gentle “U” shape. The scope was inserted gently below the inferior turbinate along the septum. The scope was advanced to visualize the adenoid structure and with small adjustments of the up/down thumb lever, the tip of the scope moved down 90 degrees to visualize the vocal cords in the pharynx. The scope was then gently pushed forward in the UES and deep into the lower esophageal sphincter (LES) and proximal stomach. The depth of the LES was noted, and the esophagus was evaluated while the patient swallowed. Esophageal specimens were obtained as indicated using a lubricated cytology brush (Kimberly‐Clark, outer diameter 1.8 mm and working length 160 cm) and forceps (Boston Scientific Radial Jaw 4 Pediatric Biopsy Forceps without Needle, outer diameter 1.8 mm and working length 160 cm). The endoscopist then readjusted his hold on the scope and lubricated the shaft further as the scope was advanced to the body of the stomach through the LES. The stomach was insufflated mildly, and the patient was encouraged to burp as needed. The pylorus was intubated by following the rugae and using the side wheel. Gastric and duodenal biopsies were obtained for histology if needed. Samples for disaccharidase assessment and small bowel bacterial overgrowth were also obtained as needed. The scope was then removed from the patient after completing the procedure (Supporting Information S1).

AEs were collected both during and after the procedure. These were classified per reported pediatric grading structure.^9^

## 3 RESULTS

**Figure 1:**
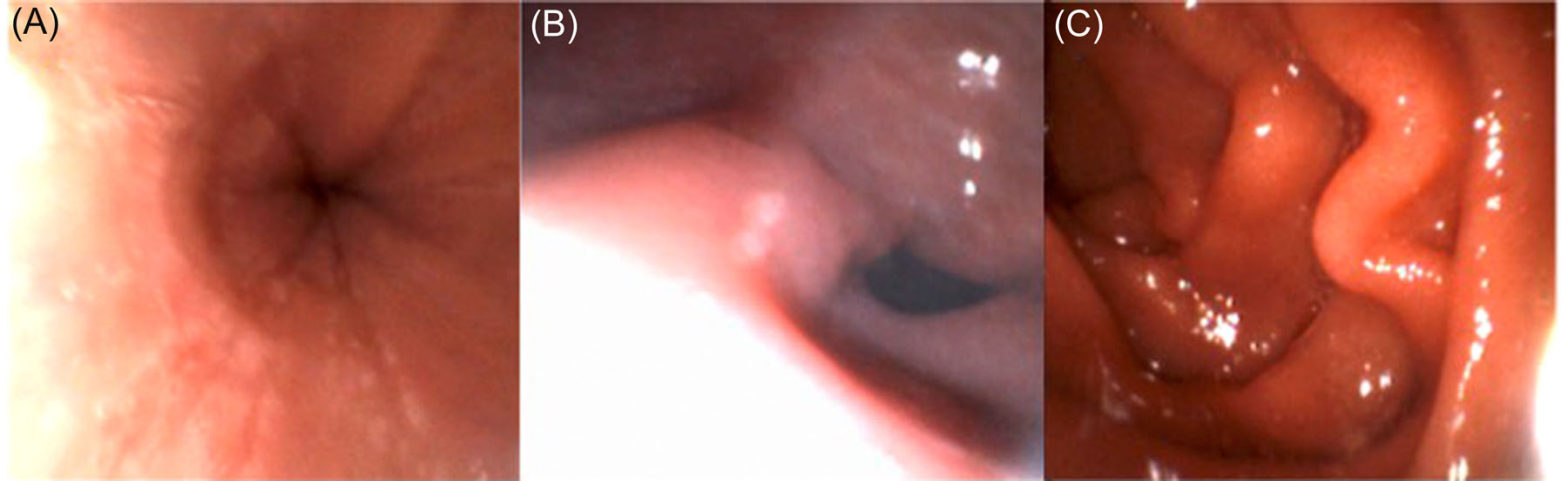
Visual findings from transnasal esophagogastroduodenoscopy. (A) Esophagus in active eosinophilic esophagitis disease. (B) Pylorus of stomach. (C) Duodenum.

Office‐based sedation‐free TNE was completed in eight patients (six males, median age: 13.5, interquartile range [IQR]: 11–15.25 years). The TNEs were performed in an outpatient clinic room designated for other GI procedures such as esophageal manometry. Procedures were performed on patients within 1–14 days after being offered. All subjects used VR and topical Lidocaine 2% gel as described. Simethicone 125 mg chewable tablets were used in two subjects and intranasal Oxymetazoline was used in two subjects. The endoscope was advanced successfully through the nares into the stomach (TN‐EG) in all subjects (100%) and into the duodenum (TN‐EGD) in seven subjects (87.5%). The only subject who did not tolerate TN‐EGD had a previous diagnosis of anxiety and she requested to abort the procedure after obtaining esophageal samples. EoE reevaluation was the indication to perform endoscopy in all subjects. Visual findings of the esophagus correlated with histologic findings (Table 1, Figure 1). Gastritis was found in four cases (50%) and duodenitis was found in two cases (25%). Biopsies were obtained from the stomach and duodenum in five subjects (62.5%) and from the esophagus in all subjects. Histology revealed patchy chronic inflammation in three out of four patients with visual gastritis and peptic duodenitis in the two patients with visual duodenitis (Table 1). Esophageal brushing for eosinophilic derived neurotoxin (EDN) measurement^10^ was performed in five subjects (62.5%). Biopsies for disaccharidase assay were obtained in one subject (12.5%). Duodenal sampling for bacterial overgrowth was obtained in one subject (12.5%). All AEs during TNE were grade 1 or lower, requiring no unanticipated medical evaluation or treatment. Notably, there were no AEs postprocedure. Two subjects (25%) experienced vomiting of a small amount during the procedure that did not require the removal of the scope. The median time from subject check‐in until discharge was 65 min (IQR: 58.75–71.25). Median time to obtain esophageal samples including esophageal brushing and two esophageal level biopsies was 6 min (IQR: 5.37–6.7). Median time to obtain duodenal biopsies was 18 min (IQR: 15.5–20.5). TN‐EGD was preferred by patients over sedated EGD in 87.5% of the cases.

**Table 1:** Subjects characteristics and findings.

						Endoscopic findings			Histological findings				
#	Age (years)	Gender	Oxymetazoline	Simethicone	Extension of study	Esophagus	Stomach	Duodenum	Esophagus (PEC)	Stomach	Duodenum	Epithelial EDN (Mcg/mL)	AEs
1	13	M	No	No	Duodenum	EREFS 4	Gastritis	Normal	25	Normal	Normal	N/A	None
2	11	M	Yes	No	Duodenum	EREFS 4	Normal	Normal	40	Normal	Normal	N/A	None
3	14	F	Yes	No	Duodenum	EREFS 2	Normal	Normal	5	Normal	Normal	N/A	None
4	20	M	No	No	Duodenum	EREFS 2	Gastritis	Duodenitis	0	Chronic patchy pnflammation	Peptic duodenitis	0.1	None
5	11	M	No	No	Duodenum	EREFS 2	Gastritis	Normal	10	Chronic patchy inflammation	Normal	2.7	None
6	15	F	Yes	No	Pylorus	EREFS 0	Normal	N/A	0	Normal	N/A	1.5	Vomiting
7	11	M	No	Yes	Duodenum	EREFS 0	Gastritis	Duodenitis	7	Chronic patchy inflammation	Peptic duodenitis	1.7	None
8	16	M	No	Yes	Duodenum	EREFS 2	Normal	Normal	60	Normal	Normal	31	Vomiting

Abbreviations: AEs, adverse events; EDN, eosinophilic derived neurotoxin (mcg/mL of esophageal brushing extract); EREFS, exudate, rings, edema, furrows and strictures; F, female; M, male; PEC, peak eosinophilic count in epithelial biopsies; TNE, transnasal endoscopy.

## 4 DISCUSSION

This is the first report of a newly released single‐use ultra slim gastroscope being used in a pediatric outpatient office‐based setting to perform complete sedation‐free TN‐EGD. In this study, we described our center's first experience in detail, and we showed that office‐based TN‐EGD is feasible with no significant AEs in the performed 8 cases. TN‐EGD was effective in monitoring disease activity because it allowed for adequate endoscopic visual and histologic assessment. It was also able to enable the testing of disaccharidases and small bowel bacterial overgrowth when indicated. The success rate to perform sedation‐free TN‐EG and TN‐EGD was 100% and 87.5% respectfully.

General anesthesia is used more commonly in pediatric endoscopy.^11^ Previous pediatric studies demonstrated risks associated with EGD under general anesthesia including cardiopulmonary and neurological complications.^12^ Pediatric studies evaluating AEs associated with EGD under anesthesia found a total AE rate of 2% and a grade 2 or higher AE rate of 1.2%.^9^ In TNE, there were no AEs that were grade 2 or higher. Vomiting was of small amount, did not require removal of the scope, and it is unlikely to increase risk of aspiration in an awake patient. Another advantage of TNE is shorter visit times for families. Time from check‐in to discharge was shorter than the average 3‐h in‐hospital time for EGD, not including time needed at home for recovery.

Using single‐use scopes may have many advantages including the ability to perform endoscopy in the office where endoscopic cleaning is not available. TNE in our center was performed in a clinic room designated for procedures rather than an ambulatory surgery center or hospital setting. The design of the single‐use ultra‐slim scope (EvoEndo) makes it feasible to complete EGDs. The scope has the capabilities of four‐way steering, suctioning, insufflation, and water perfusion. The 2.0 mm working channel allows obtaining biopsies and other samples using certain tools but, it does not allow passing other bigger tools necessary to control a gastrointestinal bleed or to resect a mass at the current time. Most endoscopists perform full EGD when reevaluating patients with EoE under anesthesia but limit their TNE practice to evaluation of the esophagus according to initial reports by Friedlander et al. For example, Nguyen et al. performed 294 TNEs but only 18 subjects (6%) had gastric biopsies and two subjects (0.6%) had duodenal biopsies collected.^3^ In this study, we performed TN‐EGD in mainly younger children and found gastritis with or without duodenitis that required a change in the management in 50% of the subjects. Epithelial EDN measured in samples obtained by pan‐esophageal brushing was performed in five cases as a complementary test to endoscopy and histology in the evaluation of EoE. It helps to overcome the unequal distribution of inflammation in EoE.

Single use endoscopes might be more costly per each single use scope, but they also save the system the required cost to clean, reprocess, and maintain/repair the reusable scopes. One may have concerns about the environmental impact of a single‐scope system. However, it should be remembered that in sedation‐free endoscopy, anesthesia waste and cleaning/reprocessing waste together is removed. The environmental impact of removing both categories of waste is not insignificant.

There were limitations to this study. This was a retrospective study performed in a pediatric setting in lower‐risk children, who were assessed by their gastroenterologist as capable of undergoing TNE to assess EoE. Thus, our findings may not be applicable to all children or adults. This cohort likely represents a highly motivated group of EoE patients and families who had undergone previous EGD under anesthesia. Additionally, an economic analysis comparison between sedation‐free endoscopy using the EvoEndo system and sedated EGD should be studied in a larger sample.

## 5 CONCLUSION

In summary, office‐based TN‐EGD with VR procedural distraction and dissociation using single‐use gastroscopes was effective to monitor EoE, gastritis, and duodenitis with no significant AEs in the performed eight cases. It allowed for biopsy, brushing, disaccharidase analysis, and bacterial overgrowth assessment. Advantages of using single use gastroscope to perform TNE in the office include decreasing risk, and cost associated with anesthesia and endoscopy suites. Because of its unique design, further consideration should be given to unsedated TN‐EGD using single‐use scopes in pediatrics, and by extension in adults, to potentially optimize disease monitoring while simultaneously decreasing cost.

## CONFLICT OF INTEREST STATEMENT

Joel A. Friedlander is Chief Medical Officer and Board Member of EvoEndo®, Inc. He is listed on numerous patents and pending patents related to endoscopic and virtual reality technologies. The remaining authors declare no conflict of interest.

## Supporting information

Supporting information.
