# Supplementary material for: Office‐based sedation‐free transnasal esophagogastroduodenoscopy with biopsies using single‐use gastroscopes: A pediatric single‐center experience
Source: JPGN Rep. 2023 Dec 20;5(1):29–34. doi: 10.1002/jpr3.12025 (PMC10964326; doi:10.1002/jpr3.12025)
Supplement: Supplementary file 1 — Supporting information. [file JPR3-5-29-s001.docx]

TN-EGD Procedure preparation and description

Procedure Preparation: Subjects referred by their gastroenterologist for TNE were given a web-based video to watch on the process of TNE (evoendo.com/patients) prior to their procedure day. Subjects were asked not to eat for 6 hours and not to drink for 2 hours before the TNE. On the day of their procedure, they met the endoscopist who reexplained the procedure, set expectations, answered questions, and obtained consents. Initial vital signs were also obtained. In an outpatient clinic room, subjects sat in a chair designed for outpatient laryngoscopy procedures. Staff were pediatric advanced life support certified, and the room had available oxygen, suction, air, and resuscitation equipment. A dedicated nurse (H.N.) helped in sending the online materials, scheduling the procedure, providing fasting instructions, and assisting during the procedure including setting up the instruments, obtaining samples and sending them to pathology or the hospital lab.

Procedure Description: The patient was positioned in the chair. Single-use video goggles with YouTube based VR programming (EvoEndo Channel) for dissociation and distraction were given. These goggles were provided in the EvoEndo patient experience kit (EvoEndo, CO, USA) and required a VR compatible Android-based phone. The patient adjusted the goggles to fit their head and chose the video to watch prior to starting the procedure. Parents were given the option to remain in the room for the TNE or to wait in another room. Simethicone 125mg chewable tablet was given orally to decrease gastric bubbles in some patients. Then, topical Lidocaine 2% gel was applied to the nasal passages (4ml) and throat (2ml). Topical Oxymetazoline was used as needed when nasal congestion was appreciated by the endoscopist. The patient held the provided stress ball in their hands, and they were asked to continue breathing and talking normally, burp as needed and vomit if necessary, during the procedure. The endoscopist engaged the patient throughout the whole procedure and explained each step. Patients were educated that the most uncomfortable part of the procedure is at the beginning during nasal passage of the scope.

All TNEs were performed by one gastroenterologist (Y.S.), and one fellow assisted for their training (J.T.). TNE was performed using a n EvoEndo® sterile, single-use, ultra-slim 3.5 mm outer diameter, 110 cm long gastroscope with 4-way deflection and a 2 mm working channel (EvoEndo, CO, USA).

The Boston Scientific Radial Jaw 4 Pediatric Biopsy Forceps without Needle (outer diameter 1.8 mm and working length 160 cm) and Kimberly-Clark cytology brush (outer diameter 1.8 mm and working length 160 cm) were used to fit in a 2.0-mm scope channel.

After connecting the scope to the controller, the scope was removed from its holder and functions were tested including white balance, suction, air insufflation, visibility, and motion. The patient was positioned so their face and nose were visualized approximately upright on the screen and the endoscopist adjusted the tip of the scope into a gentle “U” shape. The distal part of the scope was lubricated, and the tip of the scope was wiped of any lubricant material to ensure clarity of the camera. The patient was asked to blow their noses to clean any residual secretions and medication.

The endoscope was held 20 cm back from the tip to assure a smooth, gentle, and continuous insertion motion. The scope tip was parallel to the ground upon nasal entry. The scope was inserted gently below the inferior turbinate along the septum. The scope was rotated gently with small movements using the fingers and wrist mainly to achieve fine tip rotation while avoiding using the endoscope wheels. The scope was advanced to visualize the adenoid structure and with small adjustments of the up/down thumb lever, the tip of the scope moved down 90 degrees to visualize the vocal cords in the pharynx. At this point, the endoscopist paused for a few seconds to communicate with the patient and to readjust holding the shaft of the scope. The depth of the scope at the upper esophageal sphincter (UES) was noted. The endoscopist localized the UES location, prepared the patient for mild gagging during intubating the UES and asked them to swallow. The scope was then gently pushed forward in the UES and deep into the lower esophageal sphincter (LES) and proximal stomach. The endoscopist, again, paused for few seconds to congratulate the patient, assure the most uncomfortable part of the procedure was finished and to allow for swallowing and burping. The depth of the LES was noted, and the esophagus was evaluated while the patient swallowed. Additionally, both water and air were introduced gently into the lumen to obtain better visualization. Esophageal specimens were obtained as indicated using a lubricated cytology brush and forceps from different segments of the esophagus. The noted UES depth was important to maintain the scope in the esophagus and to avoid withdrawing the scope into the pharynx. After obtaining esophageal samples, the endoscopist congratulated the patient and obtained assent to proceed with gastric and duodenal intubation. The endoscopist readjusted his hold on the scope and lubricated the shaft further as the scope was advanced to the body of the stomach through the LES. The stomach was insufflated mildly, and the patient was encouraged to burp as needed. The scope was advanced and gastric secretions were suctioned until the pylorus was visualized. The pylorus was intubated by following the rugae and using the side wheel. The endoscopist also continued to assure the scope was parallel to the ground at nasal entry point for patient comfort. Gastric and duodenal biopsies were obtained for histology. Samples for disaccharidase assessment and small bowel bacterial overgrowth were also obtained as needed. The scope was then removed from the patient after completing the procedure. The patient was then brought to a consultation room, vitals were reobtained, and findings were discussed with their family. Patients were offered sips of sweet drinks and were asked to maintain clear liquids for 30 minutes. Patients were advised to resume regular activities after discharge.
